# Ultrasound-Assisted Extraction of Polyphenols from Crude Pollen

**DOI:** 10.3390/antiox9040322

**Published:** 2020-04-16

**Authors:** Mircea Oroian, Florin Ursachi, Florina Dranca

**Affiliations:** Faculty of Foond Engineering, Stefan cel Mare University of Suceava, 720229 Suceava, Romania; f.ursachi@yahoo.com (F.U.); florina.dranca@usm.ro (F.D.)

**Keywords:** pollen, polyphenol, extraction, ultrasonic

## Abstract

The aim of this study was to evaluate the extraction efficiency of polyphenols from crude pollen by an ultrasonic process. Prior to the polyphenols extraction, the crude pollen was defatted. The extraction from defatted pollen was carried out by varying four extraction parameters: ultrasonic amplitude (20%, 60% and 100%), solid/liquid ratio (10 g/L, 20 g/L and 30 g/L), temperature (35, 50 and 65 °C) and time (10, 20 and 30 min). The extracts were analyzed in terms of extraction yield (%), total phenolic content (TPC) and total flavones content (TFC). The extracted oil was analyzed in terms of fatty acids composition; myristic acid (159.1 µg × g^−1^) and cis-14-pentadecenoic acid (106.6 µg·g^−1^) were found in the highest amount in the pollen oil. The optimum conditions of extraction were determined and were, as follows: 100% amplitude of ultrasonic treatment, 30 g/L solid/liquid ratio, 40.85 °C and 14.30 min, which led to the extraction of 366.1 mg GAE/L of TPC and 592.2 mg QE/g of TFC, and also to an extraction yield of 1.92%.

## 1. Introduction

Pollen is a fine yellow-red powder-like material produced by flowering plants and is gathered by bees [1]; it results from the agglutination of flower pollen, the nectar of honey and the salivary substances from the bees [2]. The chemical composition of bee pollen depends on several factors, such as plant source, geographical origin, seasonal conditions and bee activities [3,4]. The main constituents of pollen are: proteins [2,4], carbohydrates [4,5], lipids [4,5], amino acids [4,5], polyphenols [4,6,7] and carotenoids [4,7]. Pollen composition, in mean concentrations, is made up of sugars (40%), protein (23%), including amino acids (10%) and lipids (10% reported in dry weight). The main amino acids contained by pollen are methionine, lysine, threonine, histidine, leucine, isoleucine, valine, phenylalanine and tryptophan, the main sugars are fructose (approx. 19%), glucose (approx. 15%) and sucrose (7%), while the main fatty acids are linoleic (18:2n-6), α-linolenic (18:3n-3) and palmitic (16:0) acids [2,4,5]. The color of pollen is given by the presence of the flavonoids and carotenoids (lutein, β-cryptoxanthin and β-carotene) [8].

Ultrasound-assisted extraction is a key technique that can be considered a green technology for the extraction of polyphenols from the different matrix. The extraction based on ultrasound has high reproducibility, requires lower quantities of solvents, simplifies the manipulation, eliminates the high quantities of waste water and the final extracts have higher purity [9,10,11]. The increase of extraction efficiency, alongside the reduction of the extraction time, is determined by two mechanisms: ultrasonic cavitation and mechanical mixing effect. The ultrasonic extraction mechanism involves the following processes: fragmentation, erosion, effect, sonoporation, local shear stress, and detexturation [12]. During the propagation of the ultrasounds into the solvent, the molecules’ speed increases and cell disruption appears when the cavitation bubbles collapse on the cell surface [13,14].

Ultrasound-assisted extraction of polyphenols from crude pollen was used by Yang et al. [15] and Wu et al. [16]. Yang et al. [15] used ultrasonic and ball-milling treatment to improve the extraction of bioactive compounds from rose bee pollen, while Wu et al. [16] used ultrasonic and high shear technique for improving the extraction of amino acids, fatty acids, protein, crude fat, reducing sugar, β-carotene, calcium, iron, zinc and selenium, respectively.

To our knowledge, there are no other studies related to the optimization of ultrasound-assisted extraction of polyphenols from crude pollen. The aim of this study was to investigate the ultrasonic extraction of bioactive compounds from bee pollen using a direct ultrasound-assisted extraction; the extraction parameters (ultrasonic amplitude, solid/liquid ratio, temperature) and time-input parameters were modeled using the Box–Behnken design. The extracts were analyzed in terms of extraction yield (%), total phenolic content (TPC) and total flavones content (TFC), and the fatty acid composition of pollen oil was determined using GC-MS. The extract obtained in optimal extraction conditions was analyzed in terms of individual phenolics composition (gallic acid, protocatechuic acid, vanillic acid, *p*-hydroxybenzoic acid, chlorogenic acid, caffeic acid, *p*-coumaric acid, rosmarinic acid, myricetin, quercetin, luteolin and kaempferol).

## 2. Materials and Methods

Crude pollen was purchased from a local beekeeper from Suceava County, Romania. The crude pollen was harvested in the summer of 2019 and was polyfloral. The samples were packed and stored at −20 °C until further analysis. The extraction of a lipid fraction from the pollen sample was performed by the Soxhlet method using pure hexane: 10 g of pollen was extracted with 100 mL of hexane into a Soxhlet apparatus, and the solvent was recirculated 5 times. Five batches were extracted as 10 g of crude pollen. The mixture of solvent and oil was separated using a rotary evaporator under vacuum at 65 °C. The defatted pollen was used for the extraction of polyphenols, while the lipid fraction was analyzed in terms of fatty acids composition.

Methanol, AlCl_3,_ Folin-Ciocalteau reagent, sodium carbonate, gallic acid, protocatechuic acid, *p*-hydroxybenzoic acid, caffeic acid, vanillic acid, chlorogenic acid, *p*-coumaric acid, rosmarinic acid, myricetin, luteolin, quercetin and kaempferol were purchased from Sigma–Aldrich (Taufkirchen, Germany).

### 2.1. Ultrasonic Extraction Procedure

The sample was placed in an ultrasonic bath model Ti-H-15 (Elma, Singen, Germany) at 25 kHz and a maximum power of 100 W. The extractions were carried according to the parameters presented in Table 1, using in each extract procedure 30 mL of solvent (80% (v/v) methanol).

### 2.2. Methods

#### 2.2.1. Total Phenolic Content (TPC)

The method used for the determination of total phenolic content was described by Escriche and Juan-Borras [17]. 100 µL of each extract of pollen plus 1900 µL distilled water were placed in a glass tube, and then the solution was oxidized by adding 100 µL of Folin–Ciocalteau reagent. After exactly 2 min, 800 µL of 5% sodium carbonate (w/v) was added. This solution was maintained in a water bath at 40 °C for 20 min, and then the tube was rapidly cooled with crushed ice to stop the reaction. The absorbance of the solutions was recorded at 760 nm. The TPC results were expressed as mg gallic acid equivalent/L (mg GAE/L), and all the determination were done in triplicate using methanol as blank.

#### 2.2.2. Total Flavone Content (TFC)

The method used for the analysis of total flavone content was described by Popova et al. [18]. A 2 mL aliquot of extract, 20 mL methanol and 1 mL 5% AlCl_3_ in methanol (w/v) were mixed in a volumetric flask, and the volume was made up to 50 mL with methanol. The mixture was left to rest for 30 min. The absorbance of the solutions was recorded at 425 nm. The TFC results were expressed as mg quercetin equivalent/L (mg QE/L), and all the determination were done in triplicate using methanol as blank.

#### 2.2.3. Individual Phenolics Separation and Detection

The phenolic extracts were analyzed using a high-performance liquid chromatography (HPLC) instrument (Shimadzu, Kyoto, Japan) coupled with a diode array detector. The separation was carried out on a Zorbax SP-C18 column, with 150 mm length, 4.6 mm i.d., 5 μm-diameter particle and thermostated at 25 °C. The sample volume injection was 10 µL. For the separation of phenolics, we used two mobile phases: A (0.1% acetic acid in water) and B (acetonitrile), respectively, using the following gradient: min 0 – A 100%, min 6.66 – B 5%, min 66.66 – B 40% and min 74 – B 80% [19,20,21]. The solvent flow rate was 1 mL/min. The phenolics (at 280 nm for gallic acid, protocatechuic acid, vanillic acid and *p*-hydroxybenzoic acid; and 320 nm for chlorogenic acid, caffeic acid, *p*-coumaric acid, rosmarinic acid, myricetin, quercetin, luteolin and kaempferol) were determined based on the retention times and quantified based on their calibration curves (all the curves had R^2^ higher than 0.98). For the methanoic extract, the phenolics were determined using the retention times and their nature was confirmed by their absorption spectra.

#### 2.2.4. GC Analysis of Fatty Acids Methyl Esters

Fatty acid derivation was made based on the method described by Dulf [22]. The separation of the fatty acids methyl esters was carried out on a Shimadzu GC-MS instrument (GC MS-QP 2010 Plus, Shimadzu, Japan) equipped with an AOC-01 auto-injector that was used to perform the gas chromatographic-mass spectrometric analyses. The fatty acid methyl esters were separated using a SUPELCOWAX 10 column (60 m × 0.25 mm i.d., 0.25 μm film thickness; Supelco Inc., Bellefonte, PA, USA). The initial oven temperature was 140 °C and was increased to 220 °C at a rate of 7 °C/min and then held at this temperature for 23 min. The flow rate of the carrier gas (He) and the split ratio were 0.8 mL/min and 1:24, respectively. The injector temperature was 210 °C. The mass spectrometer interface and source temperatures were 250 and 200 °C, respectively. Electroionisation mass spectra were recorded in the positive ion mode at 70 eV and with a mass range of m/z 22–395. Each measurement was made in triplicate. The injection volume was set at 1 μL. Identification of FAMEs was done by comparing their retention times with those of known standards (37 component FAME Mix, Restek, Bellefonte, PA, USA, 35077) and the resulting mass spectra to the ones from our database (NIST MS Search 2.0).

#### 2.2.5. FT-IR

Fourier-transform infrared spectroscopy (FT-IR) analysis was made using a Nicolet i-20 spectrophotometer (Thermo Scientific, Karlsruhe, Dieselstraße, Germany). The spectra were recorded in transmission mode using the Attenuated total reflectance (ATR) system within the wave number range of 4000–400 cm^−1^ at a resolution of 4 cm^−1^. SpectraGryph–spectroscopy software (Version 1.2.11, Dr. Friedrich Menges, Germany, www.effemm2.de) was used to display the spectra. The samples (crude pollen, defatted pollen, pollen extract and pollen oil) were placed on the ATR crystal, and the spectra were recorded in triplicate.

#### 2.2.6. Experimental Design and Statistical Analysis

The Box–Behnken design with four factors was used for the modeling of the pollen extraction process (Table 1). The input variables were ultrasonic amplitude, solid/liquid ratio, temperature and time and the output variables were extraction yield, TPC and TFC. Design expert 16 (trial version, StatEase, Minneapolis, MN, USA) was used for the experimental design. A second-order polynomial response was used for the pollen extraction process modeling:

(1) $$y=b_{0}+\sum_{i=1}^{n}\left(b_{i}x_{i}\right)+\sum_{i=1}^{n}\left(b_{ii}x_{ii}^{2}\right)+\sum_{ij=1}^{n}\left(b_{ij}x_{i}x_{j}\right)$$

where *y* is the predicted response (extraction yield, TFC or TPC), *x_i_* stands for the coded levels of the design variable (ultrasonic amplitude, solid/liquid ratio, temperature and time), *b*_0_ is a constant, *b_i_* = linear effects, *b_ii_* = quadratic effects and *b_ij_* = interaction effects.

## 3. Results

The influence of ultrasonic amplitude, solid/liquid ratio, temperature and time on the extraction yield, TPC and TFC is presented in Table 2.

### 3.1. Influence of Ultrasonic Amplitude

The ultrasonic amplitude had positively influenced the extraction yield (*p* < 0.001), TPC (*p* < 0.001) and TFC (*p* < 0.001); with the increase of the amplitude all three parameters increased statistically significantly. The increase of the ultrasonic amplitude led to an increase in the cavitation effects of the ultrasonics. Our findings were in agreement with those reported for the polyphenol extraction from *Nepheliuml appaceum* [23], grape seeds [24], mashed tea leaves [25] and *Acer truncatum* leaves [26]. The ultrasound waves (24–50 kHz) determined an increase in the extraction efficiency due to the cavitation process, which involved the formation and collapse of the cavitation bubbles produced in the extraction media during wave propagation. The implosion of the bubbles generated microjets and solvent flows, which in turn led to the next phenomena: cell rupture and mass transfer. These two phenomena resulted in an increase in the release of polyphenols from the matrix into the solvent.

### 3.2. Influence of Solid/Liquid Ratio

The solid/liquid ratio had a positive influence on the extraction yield (*p* < 0.001), TPC (*p* < 0.001) and TFC (*p* < 0.001); it was found that with the increase of the solid/liquid ratio all three parameters increased statistically significantly. In our study, the suitable solid/liquid ratio was 30 g/L. The knowledge regarding the optimum solid/liquid ratio is important from the economic point of view, as well as for enhancing the extraction efficiency and its outcomes. The effect of this parameter was attributed to the possibility that the reduced mixture density attained as a result of the higher solvent-to-material ratio increased the ultrasound wave propagation speed while reducing the effect of attenuation of ultrasounds and increasing the transfer of energy/distance covered by wave/time [27,28]. Our findings were in agreement with those reported by Setyaningsih et al. [29] for the extraction of polyphenols from rice grains, De Oliveira et al. [30] for the extraction of hibalactone in *Hydrocotyle umbellata* subterraneous parts and Pavlic et al. [31] for the extraction of polyphenols from peppermint.

### 3.3. Influence of Extraction Temperature

The influence of temperature on the extraction yield, TPC and TFC are presented in Table 2. As can be observed, the temperature of extraction influenced statistically significantly only the extraction yield, while in the case of TPC and TFC, the influence was not statistically significant. The increase of the extraction yield with the temperature might be because of the high solubility of different extractible compounds from pollen into methanol. The increase of temperature may increase the extraction efficiency due to the disruption of the cellular matrix structure which leads to the improvement of the solubility of polyphenols from the matrix and the mass transfer to the solvent [32]. The increase of temperature may decrease the surface tension and the viscosity, which will promote the solvent penetration into the matrix and improve the extraction process [33,34]. Our results were in agreement with those reported in the case of polyphenol extraction from wild sage [35], bioactive compounds from aromatic plants [36], polyhenols and anthocyanin from eggplant [14,37] and polyphenols from propolis [21].

### 3.4. Influence of Extraction Time

Table 2 shows the influence of time on the extraction yield, TPC and TFC. As can be observed, the extraction time had no statistically significant influence on the extraction, as only TPC showed an improvement of the concentration by 3.7%, while in the case of TFC, the concentration had decreased by 14.3%. The decrease in the TFC concentration might be because of the thermo-instability of the flavones [38]. The extraction yield was similar for all extraction times, which was due to the low particle diameter of the pollen sample (<125 µm), as we found that 10 min were enough to reach the maximum efficiency of the extraction, during which the sonication and the solvent penetrated the cell membrane and improved the mass transfer rate into the solvent [39,40].

### 3.5. Extraction Modeling

Response surface methodology was implemented via a three-block experiment Box–Behnken design to model the ultrasound-assisted extraction of bioactive compounds from crude pollen and optimize the extraction yield, TPC and TFC.

### 3.6. Extraction Yield

Experimental data for extraction yield, total phenolic content (TPC) and total flavones content (TFC) were fitted to quadratic equations using a Box–Behnken design, and the equations obtained for these parameters are presented below:

(2) $$\begin{array}{ll} \mathrm{Extraction}\;\mathrm{yield} & =1.24+0.028\cdot X_{1}+0.62\cdot X_{2}+0.03\cdot X_{3}+0.002\cdot X_{4}+0.01\cdot X_{1}^{2}+0.02\cdot X_{2}^{2}\\ & +0.02\cdot X_{3}^{2}-0.01\cdot X_{4}^{2}+0.01\cdot X_{1}\cdot X_{2}-0.02\cdot X_{1}\cdot X_{3}+0.01\cdot X_{1}\cdot X_{4}\\ & -0.003\cdot X_{2}\cdot X_{3}+0.001\cdot X_{2}\cdot X_{4}+0.02\cdot X_{3}\cdot X_{4} \end{array}$$

(3) $$\begin{array}{ll} \mathrm{TPC}=271.4+ & 36.9\cdot X_{1}+100.1\cdot X_{2}+7.7\cdot X_{3}+4.2\cdot X_{4}-19.9\cdot X_{1}^{2}-20.9\cdot X_{2}^{2}-36.9\cdot X_{3}^{2}\\ & -14.7\cdot X_{4}^{2}+27.4\cdot X_{1}\cdot X_{2}-1.4\cdot X_{1}\cdot X_{3}-11.5\cdot X_{1}\cdot X_{4}-10.6\cdot X_{2}\cdot X_{3}\\ & -2.1\cdot X_{2}\cdot X_{4}+5.2\cdot X_{3}\cdot X_{4} \end{array}$$

(4) $$\begin{array}{ll} \mathrm{TFC}=395.5- & 47.2\cdot X_{1}+175.3\cdot X_{2}-18.7\cdot X_{3}-29.1\cdot X_{4}+34.9\cdot X_{1}^{2}-29.6\cdot X_{2}^{2}-9.9\cdot X_{3}^{2}\\ & -14.5\cdot X_{4}^{2}+30.8\cdot X_{1}\cdot X_{2}+15.2\cdot X_{1}\cdot X_{3}+36.0\cdot X_{1}\cdot X_{4}-44.7\cdot X_{2}\cdot X_{3}\\ & -7.7\cdot X_{2}\cdot X_{4}+31.8\cdot X_{3}\cdot X_{4} \end{array}$$

The statistical analysis results of the three parameters using the experimental data are presented in Table 3. The second-order polynomial response surface model was used based on its adequacy to the experimental data. The statistical parameters R^2^, adj-R^2^, coefficient of variance, F-value and *p*-values were used to check the suitability of the selected model. The regression coefficient (R^2^) for extraction yield, TPC and TFC were higher than 0.93, which means that the equation had a high capacity to closely depict the predicted values to the experimental ones. The models of the studied parameters had higher F-values (534.7 for extraction yield, 11.63 for TPC and 13.73 for TFC, respectively) and low *p*-values (for all parameters the *p-value* was lower than 0.0001), which confirm the validity of the proposed model for the prediction of the parameters in function of the extraction conditions. Exactness and trustworthiness of the experiments were analyzed through the coefficient of variance. The low coefficient of variance (1.99 for extraction yield, 13.1 for TPC and 12.50 for TFC) showed good accuracy and consistency of the experiments.

### 3.7. Optimization of Extraction Parameters and Validation of the Models

A desirability function approach was used to study the adequacy, and the optimum conditions were determined and were as follows: 100% amplitude of ultrasonic treatment, 30 g/L solid/liquid ratio, 40.85 °C and 14.30 min. The extraction under optimum conditions reached 366.1 mg GAE/L of TPC, 592.2 mg QE/g of TFC and a 1.92% extraction yield.

### 3.8. Composition of Individual Phenolics

**Figure 1:**
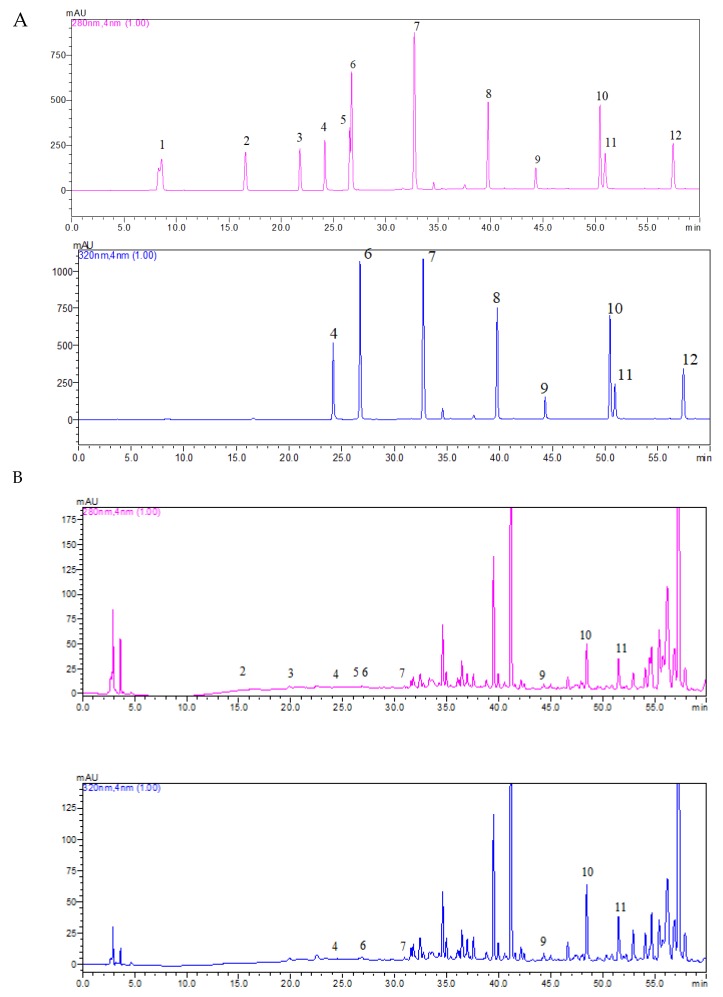
HPLC-DAD chromatogram at 280 nm and 320 nm for (**A**) standard (100 mg/l) for gallic acid–peak 1 (8.578 min), protocatechuic acid–peak 2 (16.605 min), *p*-hydroxybenzoic acid–peak 3 (21.793 min), caffeic acid–peak 4 (24.175 min), vanillic acid–peak 5 (26.547 min), chlorogenic acid–peak 6 (26.733 min), *p*-coumaric acid–peak 7 (32.733 min), rosmarinic acid–peak 8 (39.767 min), myricetin–peak 9 (44.309 min), luteolin–peak 10 (50.460 min), quercetin–peak 11 (50.947 min) and kaempferol–peak 12 (57.447 min) and (**B**) methanoic extract pollen composition.

Table 4 presents the concentrations of twelve phenolic compounds determined in the pollen extract (obtained at an ultrasonic amplitude of 100%, solid/liquid ratio of 30 g/L, 50 °C and 20 min extraction time). Figure 1 presents a typical chromatogram for a mixture of twelve phenolics and the methanoic pollen extract composition. In our study, there were determined the aglycone forms of the phenolics, but not the flavonoids glycosides and methylated flavonoids. As can be observed, the main flavonoid present was myricetin (20.54 mg/L), followed by quercetin (10.51 mg/L) and luteolin (5.79 mg/L). In terms of phenolic acids, the main compound was protocatechuic acid (6.58 mg/L), followed by chlorogenic acid (3.35 mg/L) and caffeic acid (2.41 mg/L). It would be difficult to compare our findings with other studies because there is no standardization in individual phenolics quantification to extract volume or to pollen weight. In a study regarding the polyphenols from Tunisian pollen, it was reported that the main phenolic acids were cinnamic acids (coumarin and caffeic acid) and benzoic acids (gallic acids and vanillic acids), and the common flavonoids were epicatechin, catechin, rutin and quercetin [41]. Kostic et al. [42] reported in Serbian pollen the following major compounds: 5-*O*-caffeoylquinic acids (2.54 mg/kg pollen) and caffeic acid (2.16 mg/kg) from the phenolic acids group, quercetin 3-*O*-galactoside (112.86 mg/kg) and isorhamnetin 3-*O*-glucoside (14.46 mg/kg) from the flavonols group and luteolin (1.14 mg/kg) and apigenin (0.58 mg/kg) from the flavones group. Zilic et al. [43] analyzed the composition of different flavonoids of pollen from red maize, white maize, yellow maize, blue maize, dark red maize, brown-red maize and sweet maize and observed that quercetin diglycoside was the most abundant one with concentration ranging between 31.22–45.49 mg/kg, followed by isorhamnetin diglycoside (5.18–12.99 mg/kg), hyperoside glycoside (3.93–10.12 mg/kg) and rutin derivate (3.65–6.56 mg/kg).

### 3.9. FT-IR Spectroscopy

**Figure 2:**
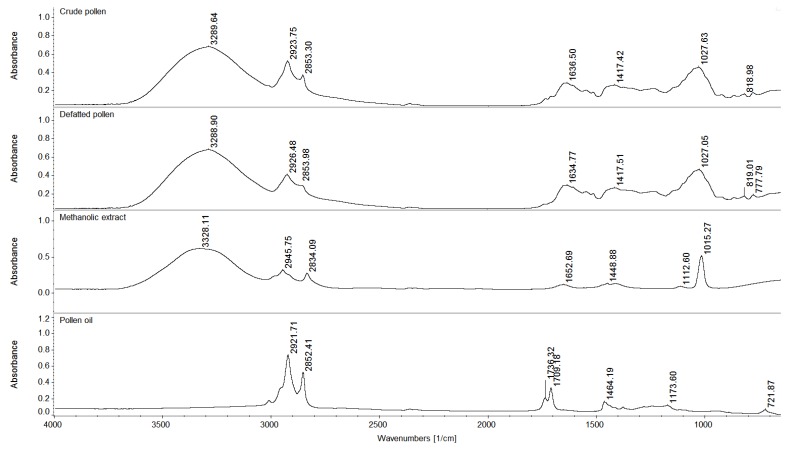
FT-IR spectra for crude pollen, defatted pollen, methanolic extract and pollen oil.

The investigation of the functional groups of samples of crude pollen, defatted pollen, pollen extract (obtained at an ultrasonic amplitude of 100%, solid/liquid ratio of 30 g/L, 50 °C and 20 min extraction time) and pollen oil was made by means of FT-IR spectroscopy. The spectra of these four samples were recorded in absorbance mode in the mid-infrared region and are presented in Figure 2. The wide absorption band, observed at 3289.64 cm^−1^ and 3288.90 cm^−1^ for crude pollen and the defatted pollen sample, and at 3328.11 cm^−1^ for pollen extract was attributed to stretching vibrations of O–H group and indicated the presence of water in the samples [44,45]. Moreover, the band at 1636.50 cm^−1^ (crude pollen) and 1634.77 cm^−1^ (defatted pollen) also corresponded to vibrations (bending) of the O–H group [45]. The presence of these bands was expected, as previous studies reported that fresh pollen had moisture content between 7% and 30% [46]. In the region 3000–2850 cm^−1^, the peaks that were identified in the spectra of the samples were assigned to symmetric and asymmetric stretching of C–H due to the presence of carbohydrates (mainly cellulose) and lipids [44,47]. For the pollen oil sample, the peaks in this region (2921.71 and 2852.41 cm^−1^) were more pronounced, which was expected considering the nature of this sample. Furthermore, the peaks at 1736.32 and 1709.18 cm^−1^, which were identified only in the absorption spectra of the oil sample, corresponded to stretching vibrations of C=O groups and were also due to the presence of lipids [48].

In the case of the crude and defatted pollen samples, the O–H vibrations around 1630 cm^−1^ were part of a broad band between 1730 and 1530 cm^−1^ which comprises C=O stretching due to both amide I and fatty acids, vibrations specific to C–N stretching and N–H deformation (amide II), and C=C stretching in unsaturated lipids and aromatic structures [44,47]. The aromatic compounds most likely include *p*-coumaric acid and ferulic acid, which are two important components of sporopollenins [49]. Absorption peaks at 1730–1530 cm^−1^ were also observed for other bee products, showing that this spectral region was the most suitable for the quantification of sugars and organic acids in honey [50] and the identification of flavonoids and amino acids in crude propolis and propolis extract [21]. The range between 1200 and 500 cm^−1^ was considered the fingerprint region for pollen, where the C–O and C–C stretching indicated differences in the saccharide, protein and lipid composition of the samples. Bands around 1030 cm^−1^ in the spectra of crude pollen and defatted pollen were assigned to stretching vibrations of saccharides and proteins, while those around 800–700 cm^−1^ were characteristic only to saccharides. For the pollen extract, the presence of protein (amide I bands) was indicated by the peak at 1652.69 cm^−1^ and that of phenolic compounds by the peaks at 1448.88 and 1112.60 cm^−1^; the peak at 1015.27 cm^−1^ corresponding to stretching of the C–O group might be determined by alcohol groups [21]. Finally, for pollen oil, the bands at 1464.19 cm^−1^ (deformation vibration of CH_2_), 1173.60 cm^−1^ (C–O stretching vibration) and 721.87 cm^−1^ (CH_2_ rocking), together with those around 1700 cm^−1^, confirmed the presence of the two main lipid groups of pollen (triglycerides and phospholipids) in this sample [50,51].

### 3.10. Fatty Acid Composition

The pollen oil fatty acid methyl esters were determined using GC-MS, and from the 37 fatty acid methyl esters examined, only 21 were quantified, which are presented in Table 5. The total concentration of fatty acids determined was 695.74 µg × g^−1^ pollen, which is similar with the concentration of fatty acids reported by Wu et al. [15] for apricot bee pollen (634.6 µg × g^−1^ pollen). The most abundant saturated acids were myristic acid (159.1 µg × g^−1^ pollen) and palmitic acid (80.51 µg × g^−1^ pollen), while the most abundant mono-saturated acids were *cis*-14-pentadecenoic acid (106.61 µg × g^−1^ pollen) and trans-9-elaidic acid 18:1ωt (106.61 µg × g^−1^ pollen), respectively. Only one polyunsaturated acid was determined as linoleic acid (11.63 µg × g^−1^ pollen). The oleic and palmitic acids play an important role in the nutrition of bees, while myristic, linoleic, linolenic acids are involved in the inhibition of growth of the spore-forming bacteria and other microbes that usually inhabit the hives [8].

In a previous study on the fatty acids from maize pollen, it was observed that the abundant saturated acids were palmitic acid and henicosanoic acid, while the most abundant unsaturated fatty acids were oleic acid and elaidic acid; linoleic acid was the most abundant polyunsaturated fatty acid as observed in our case [42]. Belina-Aldemita et al. [52] observed that the most abundant fatty acids in *Tetragonulabiroi* *Friese* from polyfloral pollen lipids were palmitic acid (28.51%), α-linolenic acid (27.66%) and linoleic acid (24.47%).

The fatty acids composition can vary from plant to plant, and in the same plant from part to part, and the same observation was made for saturated (SFAs), mono- (MUFAs) and polyunsaturated fatty acids (PUFAs). The World Health Organization [53] recommends that the ratio between UFAs and SFAs should be higher than 1.6 (WHO/FAO, 2003). The pollen oil analyzed contained 58.39% SFAs and 41.61% UFAs (39.83% MUFAs and 1.78% PUFAs), respectively; the ratio between UFAs and SFAs was 0.712, which was below the ratio recommended. Kostic et al. [42] observed that the ratio of UFAs and SFAs ranged for fatty acid of maize pollen between 0.80 to 3.70.

## 4. Conclusions

In this study, an ultrasound-assisted extraction of bioactive compounds from pollen in different conditions (ultrasound amplitude, solid/liquid ratio, temperature and time) was investigated. The ultrasound amplitude and solid/liquid ratio had a statistically significant influence on the extraction yield, TPC and TFC; the extraction temperature influence was not statistically significant for the extraction yield and TPC, while the influence of time was negligible. The extraction yield ranged between 1.20%–1.48% in function of the extraction parameters applied. A Box–Behnken design was used for extraction modeling. The main flavonoid contained in the methanolic extract (of the flavonoids under study) was myricetin (20.54 mg/L), followed by quercetin (10.51 mg/L) and luteolin (5.79 mg/L). In the case of phenolic acids, the main compounds (of the phenolic acids under study) were protocatechuic acid (6.58 mg/L), chlorogenic acid (3.35 mg/L) and caffeic acid (2.41 mg/L). The extracted oil was analyzed in terms of fatty acids composition and myristic acid (159.1 µg ·g^−1^ pollen) and cis-14-pentadecenoic acid (106.61 µg × g^−1^ pollen) were found in the highest amounts in pollen oil. The optimum conditions were determined and were, as follows: 100% amplitude of ultrasonic treatment, 30 g/L solid/liquid ratio, 40.85 °C and 14.30 min, and resulted in the extraction of 366.1 mg GAE/L of TPC, 592.2 mg QE/g of TFC and a 1.92% extraction yield.

## Figures and Tables

**Table 1:** Actual and coded values of experimental design.

Independent Variables	Coded Values		
	−1	0	1
Ultrasonic amplitude (%) – *X*_1_	20	60	100
Solid liquid ratio	10	20	30
Temperature (°C) – *X*_3_	35	50	65
Time (min) – *X*_4_	10	20	30

**Table 2:** Analysis of variance of extraction parameter influence on total phenolic content (TPC), total flavonoid content (TFC) and extraction yield.

Parameter	Ultrasonic Amplitude (%)			F Value	Solid Liquid Ratio (g/L)			F Value	Temperature (°C)			F Value	Time (min)			F Value
	20	60	100		10	20	30		35	50	65		10	20	30	
Extraction yield (%)	1.23 ^c^	1.25 ^b^	1.29 ^a^	15.4 ***	0.64 ^c^	1.25 ^b^	1.89 ^a^	7439 ***	1.24 ^b^	1.24 ^b^	1.30 ^a^	14.3 **	1.25 ^a^	1.27 ^a^	1.26 ^a^	0.08 ^ns^
TPC (mg GAE/L)	221 ^b^	222 ^b^	277 ^a^	17.5 ***	164 ^c^	233 ^b^	323 ^a^	128 ***	216 ^a^	250 ^a^	254 ^a^	0.75 ^ns^	238 ^a^	244 ^a^	247 ^a^	0.23 ^ns^
TFC (mg QE/L)	346 ^a^	359 ^a^	441 ^b^	11.4 **	197 ^c^	402 ^b^	547 ^a^	157 ***	397 ^a^	389 ^a^	360 ^a^	1.78 ^ns^	406 ^a^	392 ^a^	348 ^a^	4.34 ^ns^

GAE, gallic acid equivalent; QE, quercetin equivalent, ns - *p* > 0.05, * *p* < 0.05, ** *p* < 0.01, *** *p* < 0.001; a–c Different letters in the same column indicate significant differences among samples (*p* < 0.05).

**Table 3:** ANOVA analysis of model response for extraction yield, total phenolic content and total flavone content (TFC).

Source	DF	Extraction yield (%)		TPC (mg GAE/L)		TFC (mg QE/L)	
		F-Value	*p*-Value	F-Value	*p*-Value	F-Value	*p*-Value
Model	14.0	534.7	<0.0001	11.63	<0.0001	13.73	<0.0001
X_1_	1.00	15.4	0.0015	17.52	0.0009	11.40	0.0045
X_2_	1.00	7439	<0.0001	128.2	<0.0001	157.3	<0.0001
X_3_	1.00	14.3	0.0020	0.75	0.3998	1.78	0.2035
X_4_	1.00	0.08	0.7794	0.23	0.6390	4.34	0.0559
X_12_	1.00	0.44	0.5187	2.74	0.1198	3.36	0.0881
X_13_	1.00	2.78	0.1175	3.04	0.1030	2.42	0.1420
X_14_	1.00	5.53	0.0339	9.43	0.0083	0.27	0.6091
X_23_	1.00	1.64	0.2207	1.50	0.2413	0.58	0.4577
X_24_	1.00	0.57	0.4622	3.21	0.0946	1.62	0.2241
X_34_	1.00	1.60	0.2265	0.01	0.9273	0.40	0.5390
$X_{1}^{2}$	1.00	0.18	0.6781	0.56	0.4661	2.21	0.1590
$X_{2}^{2}$	1.00	0.06	0.8074	0.48	0.4981	3.40	0.0864
$X_{3}^{2}$	1.00	0.01	0.9110	0.02	0.8946	0.10	0.7547
$X_{4}^{2}$	1.00	3.09	0.1005	0.11	0.7398	1.72	0.2112
R^2^		0.99		0.93		0.93	
Adj-R^2^		0.98		0.84		0.86	
CV%		1.99		13.1		12.50	
Adeq.Pre		72.6		13.62		12.84	

GAE, gallic acid equivalent; QE, quercetin equivalent. R^2^, regression coefficient; Adj-R^2^, regression coefficient adjusted, CV, coefficient of variance, Adeq.Pre –adequate precision.

**Table 4:** Phenolic profiles of the optimized pollen extract using the HPLC-DAD method.

Compound	Molecular Weight	Wavelength (nm)	Retention Time (min)	Concentration (mg/L)
Gallic acid	170.12	280	8.617	ND
Protocatechuic acid	154.12	280	15.833	6.58
*p*-Hydroxybenzoic acid	138.12	280	20.686	0.73
Vanillic acid	168.14	280	25.400	0.31
Caffeic acid	180.16	320	23.103	2.41
Chlorogenic acid	354.31	320	25.605	3.35
*p*-Coumaric acid	164.05	320	31.653	2.23
Rosmarinic acid	360.31	320	39.397	ND
Myricetin	318.24	320	43.225	20.54
Luteolin	286.24	320	49.691	5.79
Quercetin	302.24	320	50.173	10.51
Kaempferol	286.23	320	56.558	ND

ND—not detected.

**Table 5:** Fatty acids composition of pollen oil.

Fatty Acid	Concentration (µg × g^−1^ pollen)
Myristic acid 14:0	159.10
*cis*-14-Pentadecenoic acid 15:1ωc	106.61
Palmitic acid 16:0	80.51
*trans*-9-Elaidic acid 18:1ωt	78.01
Hexanoic acid 6:0	46.93
Stearic acid 18:0	38.82
*z*-11-Tetradecenoic acid 14:1ωc	21.70
Pentadecanoic acid 15:0	21.20
Butyric acid 4:0	20.88
*cis*-9-Oleic acid 18:1ωc	18.05
Palmitoleic acid 16:1	15.98
Myristoleic acid 14:1	14.57
Octanoic acid 8:0	14.13
Linoleic acid 18:2ω6c	11.63
Eicosanoic acid C:20	9.68
Decanoic acid 10:0	8.60
Tridecanoic acid 13:0	8.10
11-Eicosenoic acid 20:1	6.66
Erucic acid 22:1	6.36
Heptadecanoaic acid 17:0	6.18
Lignoceric acid 24:0	2.03
